# Ervaringen en ondersteuningsbehoeften van leefstijlcoaches in het gebruik van digitale coachingsmiddelen bij cliënten met overgewicht

**DOI:** 10.1007/s12508-023-00379-w

**Published:** 2023-03-08

**Authors:** Monique Bak, Daniël Bossen, Katja Braam, Jasmijn Holla, Bart Visser, Joan Dallinga

**Affiliations:** 1 Centre of Expertise Urban Vitality, Faculteit Gezondheid, Hogeschool van Amsterdam, Amsterdam, Nederland; 2 Centre of Expertise Preventie in Zorg en Welzijn, Domein Gezondheid, Sport en Welzijn, Hogeschool Inholland, Haarlem, Nederland

**Keywords:** gecombineerde leefstijlinterventie, GLI, overgewicht, leefstijl, digitale coaching, Lifestyle intervention, Overweight, Lifestyle, Digital coaching

## Abstract

**Inleiding:**

De helft van de volwassen Nederlanders heeft matig tot ernstig overgewicht. De gecombineerde leefstijlinterventie begeleidt mensen met overgewicht naar een gezonde leefstijl. Naast fysieke contactmomenten kunnen digitale coachingsmiddelen ingezet worden om cliënten op afstand te begeleiden. In de praktijk blijkt dat digitale toepassingen nog niet ten volle worden benut. Om het gebruik te stimuleren is inzicht nodig in de ervaringen en ondersteuningsbehoeften van leefstijlcoaches ten aanzien van de inzet van digitale technologie.

**Methode:**

Met één vragenlijst en twee focusgroepgesprekken zijn data verzameld over het gebruik, de wensen en ondersteuningsbehoeften rond het inzetten van digitale coachingsmiddelen bij leefstijlcoaches. De vragenlijsten zijn descriptief geanalyseerd en de focusgroepgesprekken zijn thematisch geanalyseerd.

**Resultaten:**

Uit de vragenlijstresultaten (*N* = 79) en de focusgroepgesprekken (*N* = 10) bleek dat leefstijlcoaches vooral ervaring hebben opgedaan met videobellen, applicaties en online informatie. Ze gaven aan dat digitale coaching de zelfredzaamheid van hun cliënten ondersteunt. Online groepsbegeleiding wordt als minder effectief ervaren dan fysieke groepssessies, omdat er weinig interactie tussen cliënten plaatsvindt. Ook ervaren leefstijlcoaches praktische barrières bij het gebruik. Ze hebben behoefte aan uitwisseling van ervaringen met collega’s, scholing en instructies over de manier waarop digitale coachingsmiddelen ingezet kunnen worden.

**Conclusie:**

Leefstijlcoaches achten digitale coachingsmiddelen van toegevoegde waarde bij de individuele begeleiding van hun cliënten. Het wegnemen van praktische barrières en het faciliteren van uitwisseling en scholing kunnen een ruimere inzet van digitale coachingsmiddelen stimuleren.

**Digitaal aanvullende content:**

De online versie van dit artikel (10.1007/s12508-023-00379-w) bevat aanvullend materiaal, toegankelijk voor daartoe geautoriseerde gebruikers.

## Abstract (en)

**Introduction:**

In the Netherlands, half of the adult population is overweight. Combined Lifestyle Interventions guide overweight clients towards a healthy lifestyle. In addition to the face-to face sessions with clients, lifestyle professionals can use digital coaching tools to guide their clients remotely. In practice it appears that the digital applications are not fully used. To stimulate the use of digital technology, insight is needed into the experiences and support needs of lifestyle professionals.

**Method:**

Data about the use, wishes and support needs regarding the use of digital coaching tools among lifestyle professionals were collected by a questionnaire and two focus groups. The results of the questionnaires were analyzed descriptively and the focus groups were analyzed thematically.

**Results:**

Seventy-nine lifestyle professionals completed the questionnaire. Ten lifestyle professionals participated in a focus group. Both methods showed that professionals have gained experience with video communication, apps and online information. Lifestyle professionals mention that these digital coaching tools support the self-reliance of clients. Online group sessions are perceived as less effective than face-to-face group sessions, because of the lack of interaction between clients. Lifestyle professionals also experience practical barriers in using digital coaching tools. To stimulate the use of digital coaching tools, they need an exchange of experience with colleagues, training and instruction on how to use these tools.

**Conclusion:**

Lifestyle professionals consider digital coaching tools to be an added value to individual coaching. They see opportunities for wider use in the future when practical barriers are overcome, and exchange of experience and training are facilitated.

## Inleiding

De helft van de volwassenen in Nederland heeft matig tot ernstig overgewicht met gewichtsgerelateerde gezondheidsrisico’s [1, 2]. Als aanpak voor overgewicht is vanuit de gezondheidszorg de gecombineerde leefstijlinterventie (GLI) ontwikkeld, waarbij aandacht is voor gedragsverandering op het gebied van verschillende leefstijlfactoren, zoals voeding en beweging [3]. Een aantal GLI-programma’s wordt sinds 2019 vergoed door verzekeraars. Voor vergoeding vanuit het basispakket is een erkenning vanuit het Rijksinstituut voor Volksgezondheid en Milieu nodig. Deze GLI-programma’s worden erkende GLI’s genoemd. Deze worden gegeven door hbo-opgeleide leefstijlcoaches, onder wie diëtisten, fysiotherapeuten en oefentherapeuten, en hebben als doel overgewicht bij cliënten te verminderen. De begeleiding door de leefstijlcoach vindt face-to-face plaats via individuele en groepssessies [4].

Naast deze fysieke coaching zijn er mogelijkheden om de cliënt op afstand te begeleiden. Meerdere onderzoeken tonen aan dat coaching via digitale coachingsmiddelen, zoals een mobiele app en draagbare sensoren, een veelbelovende aanpak is om cliënten te begeleiden naar een gezonde(re) leefstijl [5–8]. De inzet van e‑health kan leiden tot verbeterde patiënttevredenheid, een toename van de therapietrouw en zelfmanagement van de cliënt [9].

Door online coaching kunnen professionals hun cliënt op afstand begeleiden bij het veranderen van gedrag [10]. Ook kan digitale communicatie de cliënt tijd besparen [11]. Daarnaast kunnen professionals de cliënt via online coaching, buiten de fysieke sessies om, van informatie voorzien over het eigen gezondheidsgedrag en de mogelijkheid bieden om in de eigen omgeving aan een gezonde leefstijl te werken [12, 13].

In dit onderzoek definiëren we digitale coachingsmiddelen als digitale middelen om cliënten te ondersteunen bij een gezonde leefstijl. We gaan hierbij uit van bestaande middelen en van specifieke programma’s die voor online coaching zijn gemaakt. Er is een verscheidenheid aan middelen die hiervoor ingezet kunnen worden, zoals web- en mobiele applicaties, draagbare sensoren, videocommunicatie en online programma’s met een spelelement [14].

In de praktijk wordt weinig gebruikgemaakt van digitale coachingsmiddelen bij de begeleiding van cliënten [15, 16]. Een van de mogelijke verklaringen hiervoor is dat het professionals ontbreekt aan de juiste kennis en digitale competenties [9]. Het gebruik van digitale coachingsmiddelen vereist een andere werkwijze van professionals [15, 16]. Daarnaast sluiten de digitale coachingsmiddelen mogelijk niet voldoende aan op de behoeften van de cliënt of beschikt de cliënt misschien niet over de benodigde digitale vaardigheden [9, 10]. Ook wordt genoemd dat er problemen zijn met de financiering van coaching met e‑health en dat toepassing van e‑health de werkdruk van professionals verhoogt [11, 17]. Ten slotte kunnen professionals terughoudend zijn bij het inzetten van digitale tools vanwege onduidelijkheid of twijfels over de zorgvuldige omgang met privacygevoelige gegevens [18].

De maatregelen tijdens de COVID-19-pandemie zijn een stimulans gebleken voor het toepassen van digitale coaching. Uit recent onderzoek blijkt dat zorgprofessionals meer zijn gaan beeldbellen en het aantal e‑consulten en patiëntportalen is toegenomen [11]. Artsen, verpleegkundigen en cliënten zijn in deze periode positiever gaan denken over digitale zorg [11]. Voor leefstijlcoaches zorgde de coronacrisis ook voor een toename in het gebruik van digitale coachingsmiddelen – cliënten werden veelal op afstand begeleid door middel van telefonische of videoconsulten [19]. Zo hebben leefstijlcoaches en cliënten ervaring kunnen opdoen met videocommunicatie. Het is echter nog niet bekend in hoeverre andere digitale coachingsmiddelen zijn toegepast en op welke manier leefstijlcoaches die inzetten, hoe ze dit ervaren en welke ondersteuningsbehoeften ze hebben.

Om het gebruik te stimuleren is inzicht nodig in de ervaringen en ondersteuningsbehoeften van leefstijlcoaches ten aanzien van de inzet van digitale technologie. Daarom richt dit onderzoek zich op de volgende vraag: wat zijn de ervaringen en (ondersteunings)wensen van leefstijlcoaches in het gebruik van digitale coachingsmiddelen bij leefstijlbegeleiding van cliënten met een gewichtsgerelateerd gezondheidsrisico?

## Methode

### Onderzoeksontwerp

In dit parallelle mixed-methods-onderzoek [20] is gebruikgemaakt van een zelfontwikkelde en vooraf geteste 18 items tellende vragenlijst over gebruik en barrières, facilitators en (ondersteunings)behoefte met betrekking tot digitale coachingsmiddelen. Aanvullend zijn focusgroepgesprekken met professionals gevoerd voor verdieping in de onderwerpen.

### Deelnemers

Voor dit onderzoek zijn professionals geïncludeerd met een aantekening voor leefstijlcoach die erkende GLI’s (Beweegkuur, Cool, SLIMMER) uitvoerden bij cliënten met overgewicht. Ook zijn professionals van niet-erkende GLI’s geïncludeerd, omdat er vooral binnen deze doelgroep deelnemers waren die ervaring hadden met online leefstijlcoaching. Daarnaast hadden de deelnemers een goede beheersing van de Nederlandse taal. Voor het vragenlijstonderzoek zijn de leefstijlcoaches geworven via partnerorganisaties van het Technologie bij Leefstijlcoaches (iTLC)-project: de Beroepsvereniging Leefstijlcoaches Nederland (BLCN), Huis voor Beweging, Visiom, de Nederlandse Vereniging van Diëtisten (NVD), FITMO, het Kenniscentrum Sport en Bewegen en LIJFSTIJL Coaches, en via sneeuwbal sampling. Aanvullend hierop zijn respondenten geworven via een video-oproep die is geplaatst op verschillende social media en websites. Via de vragenlijst konden de respondenten ook aangeven of ze wilden deelnemen aan een focusgroep. Wanneer ze hier interesse in hadden, werden ze per e‑mail benaderd en vervolgens ingedeeld in een van de twee focusgroepen, afhankelijk van beschikbaarheid. Ten behoeve van de dynamiek in de gesprekken zochten we deelnemers met uiteenlopende ervaringen met digitale coachingsmiddelen.

### Vragenlijstonderzoek

Om zo goed mogelijk aan te sluiten bij de juiste terminologie rond digitale coaching zijn de vragenlijsten vooraf getest onder leefstijlcoaches die e‑health toepassen. De vragenlijsten werden afgenomen via het online platform Qualtrics (Qualtrics XM, 2021). Wanneer de respondenten de volledige vragenlijst hadden ingevuld en hun e‑mailadres doorgaven maakten ze kans op een cadeaukaart van 50 euro. De vragenlijst (zie bijlage 1 in de digitaal aanvullende content) bestond uit 18 hoofdvragen met bijbehorende deelvragen. Deze zijn verdeeld over drie delen:

- *Demografische variabelen *(vraag 1 tot en met 6): deelnemers werd gevraagd naar leeftijd, geslacht, opleiding en werkervaring als leefstijlcoach, de doelgroep van cliënten en het type vergoeding van hun begeleidingssessies.
- *Gebruik van digitale coachingsmiddelen* (vraag 7 tot en met 12): deelnemers werd gevraagd naar (de mate van) gebruik van digitale coachingsmiddelen bij de begeleiding van cliënten met overgewicht. Deze coachingsmiddelen werden onderverdeeld in vijf categorieën: 1) videocommunicatie, 2) online video’s en online informatie, 3) interactieve mobiele apps of webapplicaties, 4) draagbare sensoren en 5) online programma’s op basis van een spelelement [12].
- *Barrières, facilitators en (ondersteunings)behoefte* (vraag 13 tot en met 18): tot slot konden respondenten aangeven welke knelpunten en bevorderende factoren zij ervaren bij de inzet van digitale coachingsmiddelen en werd de ondersteuningsbehoefte geïnventariseerd.

Bij de gesloten vragen en stellingen konden de respondenten antwoordopties selecteren. De stellingen werden op een vijfpuntslikertschaal gemeten (1 = helemaal mee eens, 5 = helemaal mee oneens). In aanvulling op de gesloten vragen en stellingen is een selectief aantal open vragen gesteld om achterliggende motivaties uit te vragen. Hierbij konden de respondenten vrije tekst invullen.

De vragenlijst is opgesteld en getest in samenwerking met partnerorganisaties van het iTLC-project. De vragenlijst werd uitgezet tussen 16 maart en 6 mei 2021. Het beantwoorden van de vragenlijsten kostte gemiddeld 15 minuten. De gegevens werden geanalyseerd met behulp van beschrijvende statistiek in SPSS, versie 26.0 (IBM SPSS, Chicago, Illinois).

### Focusgroepen

Er zijn twee focusgroepgesprekken georganiseerd. Deze gesprekken zijn online via Microsoft Teams gehouden en hadden een inductieve aanpak. De hoofdtopics waren ervaring met digitale hulpmiddelen tijdens de begeleiding, belemmerende en bevorderende factoren, en wensen en behoeften rond online begeleiding (zie bijlage 2 in de digitaal aanvullende content).

De semigestructureerde focusgroepen werden georganiseerd en begeleid door een onafhankelijke, ervaren moderator en hadden een maximale duur van 90 minuten. De moderator had geen rol in de analyse en rapportage van de onderzoeksgegevens. De gesprekken zijn opgenomen via de opnamefunctie binnen Microsoft Teams, het onlinesysteem voor beeldbellen (Microsoft Teams; Microsoft Corporation).

De resultaten van de focusgroepgesprekken zijn thematisch geanalyseerd in MAXQDA2022 [21]. Eerst werden de getranscribeerde geanonimiseerde focusgroepgesprekken gelezen door een onderzoeker (MB) om vertrouwd te raken met de data (stap 1). Vervolgens werden segmenten van de transcripten geïdentificeerd en geclassificeerd als open codes (stap 2). Terugkerende patronen werden geïdentificeerd en gesorteerd in voorlopige (sub)thema’s (stap 3). Deze thema’s werden beoordeeld door twee onderzoekers (MB en DB) en naderhand verfijnd (stap 4). Vervolgens zijn de thema’s in een discussiebijeenkomst met JD gepresenteerd, wat heeft geleid tot de definitieve opbouw en (sub)thema’s (stap 5). De uiteindelijke thema’s zijn gepresenteerd in dit artikel en het artikel is van peerreview voorzien door het hele iTLC-projectteam (stap 6).

### Ethische goedkeuring en informed consent

De ethische commissie van de Hogeschool van Amsterdam heeft een positief advies gegeven over de uitvoering van dit onderzoek (projectnummer 200718). Alle deelnemers zijn voorafgaand aan het invullen van de vragenlijst en het focusgroepgesprek geïnformeerd over het onderzoek en tekenden een informed consent. De deelnemers konden op ieder moment, zonder opgaaf van reden, beslissen om te stoppen met deelname aan het onderzoek. Bij de uitwerking van de resultaten werd de privacy van de deelnemers gewaarborgd. De gegevens zijn niet tot individuele personen te herleiden.

## Resultaten

### Deelnemers die de vragenlijst invulden

Eenentachtig respondenten hebben een start gemaakt met het invullen van de online vragenlijst. Uiteindelijk hebben 79 respondenten de vragenlijst ingevuld. De belangrijkste kenmerken van de onderzoekspopulatie zijn weergegeven in tab. 1. De gemiddelde leeftijd is 42 jaar (standaarddeviatie 12) en 81% (*n* = 64) van de respondenten is vrouw. De helft van de deelnemers (50%) heeft maximaal drie jaar werkervaring en het merendeel is hbo-geschoold (75%). De respondenten hebben een diverse opleidingsachtergrond, waarbij de meesten de opleiding Voeding & Diëtetiek hebben afgerond (27%). Het grootste deel van de cliënten is tussen de 25 en 65 jaar, waarbij de meeste leefstijlcoaches (87%) aangeven dat cliënten zich in de leeftijdsgroep van 35–50 jaar bevinden. Een kleine meerderheid van de leefstijlinterventies (52%) wordt betaald door de cliënt, gevolgd door betaling vanuit de zorgverzekering (28%).

**Table 1:** 

*geslacht, n (%)*		
vrouwen	64	(81)
mannen	15	(19)
leeftijd in jaren, gemiddelde (sd)	42	(12)
werkervaring in jaren *n* (%)		
0–1 jaar	15	(19)
1–3 jaar	24	(31)
3–10 jaar	16	(21)
10–20 jaar	16	(21)
20 jaar en meer	7	(9)
*opleidingsniveau, n (%)*		
mbo	10	(13)
hbo	59	(75)
wo	10	(13)
*opleiding, n (%)*		
voeding & diëtetiek (hbo)	21	(27)
sportkunde (hbo)	6	(8)
sport & bewegen (mbo)	6	(8)
bewegingswetenschappen (wo)	2	(3)
gezondheidswetenschappen (wo)	2	(3)
andere mbo-, hbo- of wo-opleiding	38	(48)
*Leeftijd van de doelgroep in jaren, n (% van N* *=* *79) (meerdere antwoorden mogelijk)*		
0–18 jaar	4	(3)
18–25 jaar	9	(11)
25–35 jaar	32	(41)
35–50 jaar	69	(87)
50–65 jaar	50	(63)
65- en ouder	25	(32)
*vergoeding leefstijlbijeenkomsten, n (%)*		
via de cliënt	41	(52)
via de zorgverzekeraar	22	(28)
via de ketenzorg	9	(11)
anders	5	(6)
via het bedrijf van de cliënt	2	(3)

### Deelnemers aan de focusgroepgesprekken

Er zijn twee online focusgroepgesprekken gehouden met in totaal tien deelnemers. Alle deelnemers waren vrouw, de gemiddelde leeftijd was 44 jaar (sd 7,4). Ze zijn werkzaam als leefstijlcoach en begeleiden cliënten met een verhoogd gewichtsgerelateerd gezondheidsrisico. Vier deelnemers begeleiden cliënten met een erkende GLI. De belangrijkste thema’s die we hebben geïdentificeerd zijn 1) gebruik van digitale coachingsmiddelen in de praktijk, 2) voordelen en barrières 3) online groepscoaching, 4) wensen voor het gebruik van digitale coachingsmiddelen en 5) behoefte en scholing.

### Resultaten uit de vragenlijst en focusgroepgesprekken

#### Gebruik van digitale coachingsmiddelen in de praktijk

De antwoorden van de leefstijlcoaches op de vragenlijst laten zien dat de meeste leefstijlcoaches (82%) bij de begeleiding van cliënten met overgewicht gebruikmaken van digitale coachingsmiddelen. Videocommunicatie-tools (68%), waaronder Zoom en Teams, mobiele beweeg- en voedingsapplicaties (52%), en online video’s en informatie (44%) worden het meest gebruikt. Leefstijlcoaches maken weinig gebruik van draagbare sensoren (14%), zoals een stappenteller of horloge dat het aantal minuten bewegen registreert. Slechts een paar coaches (4%) gebruikt online programma’s op basis van een spelelement, zoals een Wii Fit. De meeste leefstijlcoaches (78%) zien het als hun taak om cliënten te ondersteunen bij het gebruik van digitale coachingsmiddelen, zoals beeldbellen, apps en beweegmeters.

#### Voordelen en barrières

Het merendeel van de respondenten, 77% van de coaches, is van mening dat de inzet van digitale coachingsmiddelen de effectiviteit van hun handelen vergroot. Zo geeft 82% van de coaches aan dat digitale coachingsmiddelen de zelfredzaamheid van de cliënt bevorderen. In aansluiting op de vragenlijst geven de focusgroepdeelnemers aan vooral positief te zijn over de inzet van digitale coachingsmiddelen bij cliënten bij wie de frequentie van de contactmomenten onvoldoende is. De inzet van deze digitale coachingsmiddelen zien ze als ondersteunend, naast de fysieke bijeenkomsten. Tegelijkertijd geeft meer dan de helft (60%) van de respondenten van de vragenlijst aan dat ze tegen problemen aanlopen bij de inzet ervan. Volgens coaches vinden cliënten het gebruik van digitale coachingsmiddelen te moeilijk (43%). Daarnaast geeft bijna een derde aan (30%) dat de kosten te hoog zijn. Een ander veelgenoemde barrière (29%) is dat gebruikersinstructies vaak onduidelijk zijn geformuleerd. Negenendertig procent van de coaches noemt het bezwaar dat met digitale middelen verkregen gegevens niet automatisch in het elektronisch cliëntendossier geplaatst (kunnen) worden, waardoor het extra werk oplevert. Ook sommige deelnemers uit de focusgroep noemen dit punt.

‘Het voelt tot nu toe meer als dat ik iets extra’s moet doen, in plaats van dat het helpt de behandelingen efficiënter te maken en de behandeling te verrijken.’ (R10).

Deelnemers uit de focusgroep geven aan dat het veel tijd kost om digitale coachingsmiddelen op de juiste manier in te zetten. Deze tijd, zo geven ze aan, is niet declareerbaar bij de zorgverzekeraar.

#### Online groepscoaching

Het merendeel van de respondenten van de vragenlijst (63%) geeft aan in 2020 en 2021 tijdens de COVID-19-restricties meer gebruik te hebben gemaakt van digitale coachingsmiddelen. Dit betreft vooral videocommunicatie met een groep deelnemers. De focusgroepdeelnemers geven aan dat ze door de ervaringen tijdens de COVID-19-pandemie positiever zijn gaan denken over het gebruik van videocommunicatie:

‘Dus alles wat ik hoor qua moeilijkheid, had ik allemaal wel bedacht, maar het viel me ontzettend mee en ik vond het ook heel praktisch, in de zin van: je hoeft niet bang te zijn voor iemand in quarantaine, want de behandeling kan gewoon doorgaan. Ik vond het ook wel heel makkelijk.’ (R8)

Tegelijkertijd noemen de meeste focusgroepdeelnemers ook nadelen van online groepscoaching via beeldbellen. Zo verloopt een online groepsbijeenkomst soms wat rommeliger en is er weinig interactie tussen de cliënten. Hierdoor, zo geven ze aan, is het moeilijk om online een groepsgevoel te creëren en zijn er meer afhakers in vergelijking met de fysieke groepsbijeenkomsten. Ook ervaren sommigen dat een online groepsbijeenkomst minder geschikt is voor cliënten met beperkte digitale vaardigheden:

‘Soms hadden we echt bijeenkomsten met enorme stoorzenders doordat mensen niet weten hoe ze de microfoon op mute moeten zetten, partners die erbij komen, chats die helemaal vollopen. Kom dan nog maar eens aan coaching toe, dat lukt gewoon niet.’ (R7)

#### Wensen voor het gebruik van digitale coachingsmiddelen

Ruim de helft van de respondenten (56%) rapporteert graag meer gebruik te willen maken van digitale coachingsmiddelen. Ook de meerderheid van de focusgroepdeelnemers is van plan vaker online en offline coachen te gaan combineren. Zo wordt meermaals aangegeven dat digitale coachingsmiddelen kunnen worden gebruikt voor kennisoverdracht en dat digitale coaching tussen de fysieke bijeenkomsten in als extra begeleiding kan worden ingezet. De leefstijlcoaches vinden het echter lastig om aan te geven hoe de digitale coaching er precies uit moet zien.

‘Dat je bepaalde kennis of informatie alvast digitaal kunt aanreiken en dat je daardoor er dieper op in kunt gaan tijdens de fysieke bijeenkomst. Anders ben je heel veel tijd kwijt aan informatie die je steeds herhaalt.’ (R3)

### Behoefte en scholing

Hoewel 60% van de respondenten aangeeft over voldoende kennis en vaardigheden te beschikken, zegt ook bijna de helft (49%) behoefte te hebben aan scholing en ondersteuning. Vrijwel alle leefstijlcoaches (91%) zijn van mening dat ze tijdens hun opleiding onvoldoende geschoold zijn om digitale coachingsmiddelen in te zetten bij de begeleiding van cliënten. Volgens 41% worden te weinig trainingen gegeven gericht op het gebruik van digitale coachingsmiddelen. Ook de focusgroepdeelnemers willen ondersteuning bij de inzet van digitale coachingsmiddelen. Een meerderheid heeft behoefte aan een overzicht van digitale coachingsmiddelen die tijdens de begeleiding ingezet kunnen worden. Dit overzicht moet vooral simpel in gebruik zijn:

‘Een soort overzicht en dan het liefst ook nog met de informatie erbij van hoe betrouwbaar is dit of wat zijn de ervaringen daarmee – een meetinstrument-rating zeg maar. Dat zou me wel ideaal lijken.’ (R3)

De meerderheid van de focusgroepdeelnemers hecht veel waarde aan wat een collega of beroepsvereniging aanbeveelt. Daarnaast kan intervisie met collega’s een vorm van ondersteuning zijn om digitale tools meer te gebruiken:

‘Je kent het product niet, dan ga je je toch afvragen: is het dat dan wel waard? Als je het van iemand uit je netwerk hoort, dan is het vaak makkelijker bepalen van: oké, is het wel of niet waard om het daadwerkelijk aan te schaffen?’ (R6)

## Beschouwing

Het hoofddoel van dit onderzoek was om de ervaringen en (ondersteunings)wensen van leefstijlcoaches in het gebruik van digitale coachingsmiddelen bij leefstijlbegeleiding van cliënten met een gewichtsgerelateerd gezondheidsrisico in kaart te brengen. Vergelijkbare onderzoeken zijn gedaan bij verschillende professionals in de zorg [9, 10, 16]. Voor zover wij weten is dit onderzoek nog niet eerder verricht onder leefstijlcoaches.

Voor de begeleiding van cliënten passen leefstijlcoaches videocommunicatie het meest toe. Daarnaast worden ook apps en websites geregeld ter ondersteuning van de begeleiding gebruikt. De deelnemers van beide groepen zien voordelen in het gebruik van digitale coachingsmiddelen, zoals het bevorderen van de zelfredzaamheid van de cliënt. De deelnemers die de vragenlijst invulden en aan de focusgroepen deelnamen geven aan dat ze door de COVID-19-maatregelen meer ervaring hebben opgedaan met het gebruik van digitale coachingsmiddelen en hier positiever over zijn gaan denken. De bevindingen uit dit onderzoek komen overeen met eerder onderzoek rond dit thema en bevestigen dat zorgprofessionals sinds de COVID-pandemie meer gebruikmaken van technologie en hier ook positiever tegenover staan [11, 22, 23].

De leefstijlcoaches lopen ook aan tegen barrières bij het gebruik van online toepassingen. Zo wordt online groepsbegeleiding als minder effectief ervaren dan face-to-face groepsbegeleiding omdat er weinig interactie tussen de groepsleden plaatsvindt en non-verbaal gedrag minder goed waarneembaar is. Bevindingen uit onderzoek laten ook zien dat face-to-face dialogen tussen deelnemers meer open zijn en meer overdenking vereisen dan dialogen in een online omgeving [24]. Daarnaast kan het gebruik van digitale coachingsmiddelen meer tijd kosten en ontbreekt het vaak aan een koppeling met cliëntendossiers. Deze bevindingen worden bevestigd vanuit de Nederlandse e‑healthmonitor 2021, waarbij zorgprofessionals als nadeel aangeven dat digitale communicatie de werkdruk verhoogt [11]. Verder blijkt dat deze vorm van coaching te moeilijk wordt bevonden voor minder digitaalvaardige cliënten. Het onderzoek van Kloek et al. laat zien dat de digitale vaardigheden van de cliënt een belangrijke rol spelen bij het inzetten van digitale coachingsmiddelen [9]. Daarbij gaat het vooral om het lerend vermogen en niet zozeer om de vraag of de cliënt al bij aanvang over voldoende of onvoldoende digitale vaardigheden beschikt. Hierbij is dus een ondersteunende rol voor de leefstijlcoach weggelegd. Dit sluit aan op onze resultaten: de meerderheid van de leefstijlcoaches ziet deze begeleiding ook als een taak van de leefstijlcoach.

De meeste leefstijlcoaches vinden dat ze bekwaam zijn in het toepassen van digitale coachingsmiddelen. Toch is er behoefte aan ondersteuning door middel van scholing. Dit sluit aan bij het onderzoek van Houwink et al., die aangeven dat het implementeren van e‑health in onderwijs essentieel is voor het toepassen ervan in de praktijk [25]. Uit de focusgroepgesprekken wordt ook duidelijk dat het voor de inzet van digitale coachingsmiddelen belangrijk is wat peers en de beroepsvereniging aanbevelen.

### Sterke punten en beperkingen

Voor onze rapportage waren de thema’s die voortkwamen uit de thematische analyse bepalend. Dit betekent dat we niet alle vragenlijstresultaten in de resultaten hebben opgenomen. Een sterk punt van dit onderzoek is dat we gebruik hebben gemaakt van een parallel mixed-methods-onderzoeksontwerp, waarbij vragenlijsten en interviews zijn gecombineerd. Dit heeft de betrouwbaarheid van ons onderzoek vergroot. Ook stemmen de resultaten uit beide onderzoeken sterk overeen. Een beperking van dit onderzoek is dat de grootte en de kenmerken van de bronpopulatie niet helemaal te achterhalen zijn en dat er mogelijk sprake is van selectiebias. Hierdoor kunnen we niet met zekerheid vaststellen of de bevindingen representatief zijn voor alle leefstijlcoaches. Daarnaast waren we door de COVID-19-maatregelen genoodzaakt om de focusgroepen online te organiseren. Dit heeft invloed gehad op de groepsdynamiek en interactie tussen de deelnemers.

### Implicaties voor de praktijk en onderzoek

Onze bevindingen laten zien dat leefstijlcoaches digitale coachingsmiddelen als toegevoegde waarde zien voor de leefstijlbegeleiding van cliënten, bijvoorbeeld doordat ze de zelfredzaamheid van de cliënt vergroten. Digitale coaching vervangt hierbij niet de fysieke contactmomenten, maar vormt een aanvulling hierop. Zo kan ze worden ingezet als extra ondersteuning tussen de fysieke contactmomenten. Een kanttekening kan worden gemaakt bij de online groepssessies. Deze slaan onvoldoende aan bij de leefstijlcoaches. Ons advies is dan ook deze bijeenkomsten zo veel mogelijk face-to-face te houden.

Voor implementatie van digitale coachingsmiddelen in de praktijk moet ook worden gekeken naar barrières, zoals de tijd die het de leefstijlcoach kost om deze middelen in te zetten, die bovendien niet declareerbaar is, en het ontbreken van een koppeling met het elektronisch patiëntendossier (EPD). Een aanbeveling is om het gebruik van digitale coachingsmiddelen te vergoeden door bijvoorbeeld naar de mogelijkheden van een keten diagnose-behandelcombinatie (dbc) te kijken. Hierbij wordt voor één cliënt één totaalbedrag uitgekeerd. De professional krijgt zo tijdens het GLI-traject meer regie over de toepassing van e‑health. Ook kan het integreren van applicaties in het EPD mogelijk drempelverlagend werken. Daarnaast kunnen de e‑healthcompetenties van leefstijlcoaches worden vergroot door een gerichte scholing. Hierbij zou onder andere aandacht moeten zijn voor het vergroten van kennis over e‑healthapplicaties, veiligheidskwesties, ethiek en het leren omgaan met de digitale vaardigheden van de cliënt [25].

## Conclusie

Onze bevindingen laten zien dat leefstijlcoaches tijdens de begeleiding van de cliënt vooral gebruikmaken van videobellen, applicaties en online informatie. Het gebruik van digitale coachingsmiddelen kan volgens leefstijlcoaches de zelfredzaamheid van de cliënt bevorderen en de effectiviteit van het handelen van de coach vergroten. Online groepssessies worden als minder effectief ervaren dan fysieke groepssessies. Een succesvolle implementatie vraagt enerzijds dat het inzetten van deze digitale coachingsmiddelen weinig tijd kost of dat deze tijd wordt vergoed, de efficiëntie vergroot en kan worden geïntegreerd in bestaande systemen. Anderzijds zijn er kansen door gerichte scholing. Ook kunnen collega’s of de beroepsvereniging een rol spelen bij de ondersteuning door intervisie, waarin ze met elkaar kunnen sparren over de mogelijkheden, het nut en de noodzaak van de inzet van digitale coachingsmiddelen.
