# Supplementary material for: Experiences and support needs of lifestyle professionals in the use of digital coaching tools for clients with overweight
Source: TSG. 2023 Mar 8;101(2):38–45. [Article in Dutch] doi: 10.1007/s12508-023-00379-w (PMC9994399; doi:10.1007/s12508-023-00379-w)
Supplement: Supplementary file 1 [file 12508_2023_379_MOESM1_ESM.docx]

**Bijlage 1. Vragenlijst digitale coaching bij leefstijlprofessionals**

**Algemene gegevens**

1. Wat is je geslacht?

- Man
- Vrouw
- Overig

2. Welke mbo- hbo- en of universitaire opleiding heb je voltooid?

- Sport en bewegen (mbo)
- Voeding & Diëtetiek (hbo)
- Fysiotherapie (hbo)
- Oefentherapie (hbo)
- Sportkunde (hbo)
- Andere mbo opleiding, namelijk..
- Andere hbo opleiding, namelijk
- Bewegingswetenschappen (universiteit)
- Gezondheidswetenschappen (universiteit)
- Andere universitaire opleiding, namelijk..

3. Hoe lang voer je al werkzaamheden uit waarin je cliënten begeleid naar een

gezondere leefstijl (in jaren)?

- ……jaren

4. Wat is je leeftijd in jaren?

- ----- jaren

5. Hoe worden leefstijlbijeenkomsten met de cliënt vergoed? (meerdere antwoorden mogelijk)

- Via zorgverzekeraar
- Via die cliënt zelf
- Via het bedrijf waar de cliënt werkzaam is
- Via ketenzorg
- Anders, namelijk……

6. Welke leeftijdsgroep zie je voornamelijk tijdens je werkzaamheden als leefstijlprofessional? (meerdere antwoorden mogelijk).

- 0 tot 12 jaar
- 12 tot 18 jaar
- 18 tot 25 jaar
- 25 tot 35 jaar
- 35 tot 50 jaar
- 50 tot 65 jaar
- 65 tot 75 jaar
- 75 jaar of ouder

**Digitale coachingsmiddelen**

Hieronder staan vragen over digitale coachingsmiddelen zoals mobiele apps om cliënten te motiveren om meer te bewegen of videobellen om cliënten op afstand te begeleiden.

7. Gebruik je op dit moment digitale coachingsmiddelen bij de begeleiding van cliënten met overgewicht?

- Ja, ik begeleid mijn cliënt volledig online
- Ja, ik gebruik digitale middelen tijdens of naast de fysieke bijeenkomsten met cliënten
- Nee, ik gebruik geen digitale middelen tijdens of naast de fysieke bijeenkomsten met cliënten

8. Kruis aan welke digitale coachingsmiddelen je op dit moment gebruikt binnen de werkzaamheden om cliënten te ondersteunen bij een gezond gedrag (meerdere antwoorden mogelijk).

- Het gebruik van online video’s en online informatie. *Bijvoorbeeld de website Thuisarts.nl of YouTube met video’s van beweegoefeningen.*
- Videocommunicatie. *Bijvoorbeeld begeleiding en coaching via de beeldbelprogramma’s Skype of Teams*
- Een interactieve mobiele app of webapplicatie. *Bijvoorbeeld een app met een programma die deelnemers aanzet om gezonde voedingskeuzes te maken*
- Draagbare sensoren. *Bijvoorbeeld een stappenteller of horloge die het aantal minuten bewegen registreert*
- Online programma met een spelelement*. Bijvoorbeeld de Wii Fit die een spelelement gebruik om spelers te stimuleren tot meer bewegen.*
- Anders, namelijk…
- Geen toepassingen

***Let op: vr 8a- 8e indien van toepassing op basis van de antwoorden van vraag 8***

8a.Welke online video's en of informatie pas je toe in de praktijk?

8b. Welk programma gebruik je in de praktijk voor videocommunicatie?

8c. Welke mobiele- en/of webapplicaties gebruik je in de praktijk om cliënten te ondersteunen in een gezonde leefstijl?

8d. Welke draagbare sensoren gebruik je in de praktijk om cliënten te ondersteunen in een gezonde leefstijl?

8e. Welk online programma op basis van spelelement gebruik je in de praktijk om cliënten te ondersteunen in een gezonde leefstijl?

***Let op: vr 9 indien van toepassing op basis van de antwoorden van vraag 8***

9. Hoe vaak gebruik je de digitale coachingsmiddelen bij de begeleiding van cliënten met overgewicht?

- Minder dan 1x per maand
- Minder dan 1x per week
- 1 of meerdere keren per week
- Dagelijks
- Bij elk contactmoment

***Let op: vr 10 indien van toepassing op basis van de antwoorden van vraag 8***

10. In welke fase van het coachtraject gebruik je de onderstaande digitale coachingsmiddelen (meerdere antwoorden mogelijk)?

- Bij de intake/voorafgaand de interventie
- Als informatiebijeenkomst voorafgaand aan de leefstijlbijeenkomst
- Tijdens een leefstijlbijeenkomst
- Na een leefstijlbijeenkomst

11a. Maak je door de COVID-19 maatregelen meer gebruik van e-coaching in de werkzaamheden als leefstijlcoach?

- Ja, omdat …
- Nee > ga door naar vraag 12

11b.Welke digitale coachingsmiddelen gebruik je tijdens de COVID-maatregelen meer dan voorheen? (meerdere antwoorden mogelijk)

- Online video's en informatie
- Videocommunicatie
- Interactieve mobiele app of webapplicatie (zoals een patiëntenportaal)
- Draagbare sensoren
- Een online programma met een spelelement
- Anders, namelijk.....

12. Zou je meer gebruik willen maken van digitale coachingsmiddelen in de begeleiding van cliënten met overgewicht?

- Ja, omdat….
- Nee
- Weet ik niet

12a. Welke digitale coachingsmiddelen zou je graag meer willen gebruiken bij de begeleiding van cliënten met overgewicht?

- Online video’s en online informatie, zoals Thuisarts.nl of YouTube video’s met beweegoefeningen
- Videocommunicatie. *Bijvoorbeeld begeleiding en coaching via beeldbelprogramma’s zoals Skype, Zoom, Teams*
- Interactieve mobiele app of webapplicatie. *Bijvoorbeeld een app met een programma die deelnemers aanzet om gezonde voedingskeuzes te maken of een patiëntenportaal*
- Draagbare sensoren. *Bijvoorbeeld een stappenteller of horloge die het aantal minuten bewegen registreert*
- Online programma op basis van spelelement. *Bijvoorbeeld de Wii Fit die een spelelement gebruik om spelers te stimuleren tot meer bewegen*
- Anders, namelijk

**Stellingen over digitale coachingsmiddelen**
De onderstaande stellingen gaan over de inzet van digitale coachingsmiddelen tijdens de leefstijlbegeleiding van cliënten met overgewicht. Geef aan in hoeverre je het eens bent met de onderstaande uitspraken.

13a. Ik beschik over voldoende kennis en vaardigheden om digitale coachingsmiddelen te gebruiken

- Helemaal mee eens
- Mee eens
- Neutraal
- Mee oneens
- Helemaal mee oneens

13b. Tijdens mijn opleiding ben ik voldoende geschoold in het gebruik van digitale coachingsmiddelen

- Helemaal mee eens
- Mee eens
- Neutraal
- Mee oneens
- Helemaal mee oneens

13c. Ik heb behoefte aan scholing en ondersteuning voor het gebruik van digitale coachingsmiddelen

- Helemaal mee eens
- Mee eens
- Neutraal
- Mee oneens
- Helemaal mee oneens

***Vervolg op 13c bij antwoorden ‘helemaal mee eens’ of ‘mee eens’***

Je hebt aangeven behoefte te hebben aan scholing en ondersteuning voor het gebruik van digitale coachingsmiddelen. Waar heb je behoefte aan?

13d. Ik laat het gebruik digitale coachingsmiddelen liever aan mijn collega's over

- Helemaal mee eens
- Mee eens
- Neutraal
- Mee oneens
- Helemaal mee oneens

13e. Ik heb het geloof dat de inzet van digitale coachingsmiddelen de effectiviteit van mijn behandeling vergroot.

- Helemaal mee eens
- Mee eens
- Neutraal
- Mee oneens
- Helemaal mee oneens

13f. Het gebruik van digitale coachingsmiddelen geeft mij meer voordelen dan nadelen

- Helemaal mee eens
- Mee eens
- Neutraal
- Mee oneens
- Helemaal mee oneens

***Vervolg op 13f*** , ***bij antwoorden ‘helemaal mee eens’ of ‘mee eens’***

Je hebt aangegeven dat het gebruik van digitale coachingsmiddelen meer voordelen geeft dan nadelen. Kun je aangeven welke voordelen dit zijn?

13g. Ik zit niet te wachten op de komst van (nieuwe) digitale coachingsmiddelen

- Helemaal mee eens
- Mee eens
- Neutraal
- Mee oneens
- Helemaal mee oneens

13h. Het gebruik van digitale coachingsmiddelen kan tijd en kosten besparen

- Helemaal mee eens
- Mee eens
- Neutraal
- Mee oneens
- Helemaal mee oneens

13i. Ik ben bang om mijn werk of een deel van (de inhoud van) mijn werkzaamheden als leefstijlcoach kwijt te raken door de komst van (nieuwe) digitale coachingsmiddelen

- Helemaal mee eens
- Mee eens
- Neutraal
- Mee oneens
- Helemaal mee oneens

13j. Het gebruik van digitale coachingsmiddelen kan de werkdruk verhogen

- Helemaal mee eens
- Mee eens
- Neutraal
- Mee oneens
- Helemaal mee oneens

13k. Het aanbod van bruikbare digitale coachingsmiddelen is voldoende voor de begeleiding van mijn cliënten

- Helemaal mee eens
- Mee eens
- Neutraal
- Mee oneens
- Helemaal mee oneens

13l. Cliënten zitten niet te wachten op de komst van (nieuwe) digitale coachingsmiddelen

- Helemaal mee eens
- Mee eens
- Neutraal
- Mee oneens
- Helemaal mee oneens

13m. Ik vind het mijn taak om cliënten te ondersteunen bij het gebruik van digitale middelen, zoals beeldbellen, apps en beweegmeters

- Helemaal mee eens
- Mee eens
- Neutraal
- Mee oneens
- Helemaal mee oneens

13n. Ik ben bang dat het gebruik van digitale coachingsmiddelen leidt tot minder persoonlijk contact met cliënten

- Helemaal mee eens
- Mee eens
- Neutraal
- Mee oneens
- Helemaal mee oneens

13o. Het gebruik van digitale coachingsmiddelen kan bijdragen aan de zelfredzaamheid van cliënten

- Helemaal mee eens
- Mee eens
- Neutraal
- Mee oneens
- Helemaal mee oneens

**Knelpunten rondom gebruik**

14. Zijn er problemen waar je tegenaan loopt bij het gebruik van digitale

coachingsmiddelen tijdens de leefstijlbegeleiding?

- Ja, namelijk …
- Nee

**15.** Wat zijn volgens jou de belangrijkste factoren die het gebruik van digitale coachingsmiddelen in de weg (kunnen) staan? Kruis de belangrijkste 5 factoren aan.

- **Digitale middelen zijn niet gebruiksvriendelijk**
- **Digitale middelen zijn onvoldoende beschikbaar**
- **Digitale middelen zien er niet aantrekkelijk uit**
- **De gebruikersinstructies zijn vaak onduidelijk**
- Data worden niet automatisch in cliëntendossiers ingevoerd en leveren daarom extra werk
- **Er wordt te weinig training gegeven aan leefstijlcoaches voor het gebruik van digitale middelen**
- **Er wordt te weinig training gegeven aan cliënten voor het gebruik van** digitale middelen
- **Er treden te vaak technische problemen in de praktijk**
- **Het gebruik past niet in de werkprocessen van leefstijlprofessionals**
- **Het gebruik sluit niet aan op de behoefte van cliënten**
- **Cliënten vinden het te moeilijk**
- **De privacy is onvoldoende gewaarborgd**
- **De kosten van e-coach zijn te hoog**
- **Anders, namelijk………….**

**Gebruik van digitale coachingsmiddelen in de praktijk**

**16.** Welke kennis en vaardigheden zijn volgens jou het belangrijkst om het gebruik van digitale coachingsmiddelen onder leefstijlprofessionals te bevorderen?

**17.** Stel we ontwikkelen producten om het gebruik van digitale coachingsmiddelen voor leefstijlprofessionals te bevorderen. Hoe zouden we dit het beste kunnen aanbieden? (meerdere antwoorden mogelijk)

- Een handleiding
- Een video met informatie en aanbevelingen
- Een webinar of scholing
- Voorbeelden van best practices
- Weet ik niet
- Anders, namelijk ________________________________________________

18. Wat zijn volgens jou de gevolgen van COVID-19 voor het toekomstige gebruik van digitale coachingsmiddelen door leefstijlprofessionals?

**19.** Naast deze digitale vragenlijst willen we ook enkele (online) focusgroepgesprekken afnemen. Tijdens deze gesprekken zullen we meer doorvragen op de ervaringen en behoeften die je hebt ten aanzien van digitale coachingsmiddelen. Deze groepsinterviews duren ongeveer 60 minuten en je ontvangt een vergoeding van 20 euro voor deelname. Mogen wij jou hiervoor benaderen? (dit wordt niet voor andere doeleinden gebruikt).

- Ja, mijn e-mailadres is…….
- Nee
