# Supplementary material for: Experiences and support needs of lifestyle professionals in the use of digital coaching tools for clients with overweight
Source: TSG. 2023 Mar 8;101(2):38–45. [Article in Dutch] doi: 10.1007/s12508-023-00379-w (PMC9994399; doi:10.1007/s12508-023-00379-w)
Supplement: Supplementary file 2 [file 12508_2023_379_MOESM2_ESM.docx]

Bijlage 2. Topiclijst focusgroep

**1.Eerste indruk digitale coaching**

- *Wat komt er het eerste in u op als je aan digitale coaching denkt?*

**2.Ervaringen en belemmeringen rondom inzet digitale coaching**

- *Wat zijn u ervaringen omtrent inzet digitale coaching?*
- *Wat gebruikt u? Waarom wel/niet?*
- *Ervaren jullie bepaalde belemmeringen omtrent inzet digitale coaching?*
- *Welke zijn dat dan?*
- *Waarom zijn dat belemmeringen?*

**3.Ondersteuningsbehoeften en wensen rondom inzet digitale coaching**

- *Heeft u behoefte aan ondersteuning omtrent digitale coaching?*
- *Zo ja, waarin zou u ondersteund worden omtrent digitale coaching?*
- *Wat is er nodig om digitale coaching wel toe te kunnen passen/om de belemmeringen weg te halen?*
- *Zou u in de toekomst (nog meer) digitale coaching willen toepassen?*
- *Hebben jullie naast de ondersteuningsbehoeften nog wensen ten aanzien van digitale coaching? Zo ja, welke?*
- *Hoe kan dit bewerkstelligd worden? (met behulp van een handleiding, informatie/aanbevelingen, video, webinar/scholing, voorbeelden van best practice, anders…?).*
